# Firmness Perception Influences Women’s Preferences for Vaginal Suppositories

**DOI:** 10.3390/pharmaceutics6030512

**Published:** 2014-09-10

**Authors:** Toral Zaveri, Rachel J. Primrose, Lahari Surapaneni, Gregory R. Ziegler, John E. Hayes

**Affiliations:** 1 Sensory Evaluation Center; The Pennsylvania State University, University Park, 16801 PA, USA E-Mails: tzz1@psu.edu (T.Z.); rjp27@psu.edu (R.J.P.); 2 Department of Food Science, College of Agricultural Sciences, The Pennsylvania State University, University Park, 16801 PA, USA; E-Mails: lus29@psu.edu (L.S.); grz1@psu.edu (G.R.Z.)

**Keywords:** microbicide, acceptability, product optimization, sensory perception, semisoft suppository, tenofovir

## Abstract

Microbicides are being actively researched and developed as woman-initiated means to prevent HIV transmission during unprotected coitus. Along with safety and efficacy, assessing and improving compliance is a major area of research in microbicide development. We have developed carrageenan-based semisoft vaginal suppositories and have previously evaluated how physical properties such as firmness, size and shape influence women’s willingness to try them. Firmness has previously been quantified in terms of small-strain storage modulus, G’, however large-strain properties of the gels may also play a role in the firmness perception. In the current study we prepared two sets of suppositories with the same G’ but different elongation properties at four different G’ values (250, 2500, 12,500, 25,000 Pa): For convenience we refer to these as “brittle” and “elastic”, although these terms were never provided to study participants. In the first of two tests conducted to assess preference, women compared pairs of brittle and elastic suppositories and indicated their preference. We observed an interaction, as women preferred brittle suppositories at lower G’ (250, 2500 Pa) and elastic ones at a higher G’ (25,000 Pa). In the second test, women evaluated samples across different G’, rated the *ease-of-insertion* and *willingness-to-try* and ranked the samples in order of preference. Brittle suppositories at G’ of 12,500 Pa were most preferred. *In vitro* studies were also conducted to measure the softening of the suppositories in contact with vaginal simulant fluid (VSF). Release of antiretroviral drug tenofovir in VSF was quantified for the brittle and elastic suppositories at G’ of 12,500 Pa to determine the effect of suppository type on release. The initial rate of release was 20% slower with elastic suppositories as compared to brittle suppositories. Understanding how different physical properties simultaneously affect women’s preferences and pharmacological efficacy in terms of drug release is required for the optimization of highly acceptable and efficacious microbicides.

## 1. Introduction

Microbicides are being developed as a pre-exposure prophylaxis of human immunodeficiency virus (HIV) and other sexually transmitted infections (STIs) [1,2,3]. A number of microbicide candidates are currently in clinical trials but a clinical impact is yet to be realized, as several promising candidates have shown mixed results. For example, 1% tenofovir (TFV) gel is being investigated in several clinical trials, of which some have shown efficacy [1], while others not [4]. Participant adherence to the study protocol appears to be a major factor governing the efficacy of microbicide candidates in clinical trials [5]. This is reflected in the CAPRISA 004 trial results, where women who used a TFV gel in more than 80% of their sex acts showed a 54% reduction in HIV infections, whereas women who used the gel in less than half of their sex acts had only a 28% reduction [1]. Subsequently, the VOICE trial studying the efficacy of a TFV gel had the gel study arm discontinued due to lack of efficacy [4]. In a detailed evaluation of patient compliance, only 36% of women assessed had actually used the gel within the week, despite 90% reporting to do so [6]. These data suggest user adherence is a critical factor governing microbicide success, and formulations, while biologically efficacious, are effective only if used as directed. Accordingly, a number of methods are being investigated to track and/or improve adherence to microbicide use during clinical trials including daily reporting using interactive voice response systems [7,8] and imaging of used applicators [9].

Varied studies have been conducted to understand product attributes that influence acceptability and thus adherence [10,11]. Several studies have explored how physical attributes of products and rheological properties can influence women’s perceived efficacy [12,13], which, in turn affects acceptability. These product attributes include appearance [14,15], smell [14,15], taste, and textural properties that may affect sexual pleasure (how the product feels during intercourse) [11,12] and leakage (the propensity of the product to seep out of the body) [10,12,14,16], as well as vaginal coating [12]. Our group has previously conducted focus groups to assess how physical properties such as size, shape and firmness of suppositories developed in the lab influence women’s perception of efficacy and willingness to try [15]. During our study, in spite of being instructed that different sizes would contain the same amount of medication, bigger sizes were perceived to be more effective, especially against HIV [15]. One of the primary drivers for selection of a particular size, shape or firmness was the perceived ease of insertion, which also influenced their willingness to try the product [15]. Li *et al.* [17] showed a high correlation between the anticipated *ease-of-insertion* and *willingness-to-try* ratings, suggesting physical characteristics of the drug carrier are an equally important consideration in the design of effective microbicides.

To address different needs and preferences of women, microbicides are being developed in different physical forms: Tablets [18], quick dissolving films [19,20], gels [21,22] and removable rings [23,24]. These choices are rheologically diverse and include products that dissolve within the body, eventually being discharged, as well as products that need to be removed and replaced periodically. Notably, many so-called “gels” in the literature are not gels in a technical sense, as they are highly viscous non-Newtonian liquids, not solids. Recently, we have developed microbicide prototypes that fall between the two extremes of tablets and viscous liquid “gels” to improve upon negative attributes that have been reported with these existing technologies. For example, leakage has been cited as one of the drawbacks of liquid “gels” [25], while the dissolution of tablets creates a hypertonic solution. Ideally, our prototypes would disintegrate inside the body and be eliminated with vaginal mucus secretions. Leveraging soft-gel technology, our delivery system consists of semisoft vaginal suppositories prepared from carrageenan, which has several advantages over gelatin, the traditional soft-gel matrix. Use of carrageenan helps circumvent negative aspects of gelatin, such as the risk of zoonotic infections, the lack of acceptability by vegetarians and the insufficient heat stability for storage in tropical climates. Carrageenan also has a distinct advantage because of its reported anti-viral activity against viruses other than HIV [26,27,28]. We have previously conducted acceptability studies with gel suppository prototypes and identified physical attributes (size, shape and firmness) and user preferences (frequency of application and duration of protection) most favorable to women [15,17,29]. In this iterative design process to identify physical characteristics that influence perceived *ease-of-insertion* and *willingness-to-try*, we have developed products across a range of firmness, size and shape. The firmness of the prototype gels used to prepare the suppositories was characterized by the storage modulus (G’) of the gels at 25 °C and ranged from 250 to 25,000 Pa. The size of the products varied from 1 to 5 mL and shapes included both standard (long oval, tampon) and non-standard (spherical) shapes for suppositories. Using this approach, we have identified preferred shapes, size and firmness for the microbicide prototypes using mixed methods (e.g., qualitative focus groups and large scale quantitative *ex vivo* consumer acceptability trials [1,15,17,29]).

In our prior work, suppositories, especially those at the lower firmness (G’), would often break while being manipulated in the hand, causing women to consider them difficult to insert. To overcome this, we reformulated the suppositories so they could be deformed to a greater extent before fracturing. The current study was designed to investigate whether suppositories with the same physical firmness (defined in terms of storage modulus, G’) but with different elongation properties influence women’s imagined *ease-of-insertion* and thus firmness preferences.

## 2. Experimental Section

### 2.1. Materials

Commercial samples of κ-carrageenan (Gelcarin NF 911, batch number: 10,707,011) and ι-carrageenan (Gelcarin NF 379, batch number: 10,514,011 were kindly provided by FMC Biopolymers (Philadelphia, PA, USA). Tenofovir (TFV) was kindly provided by Gilead Sciences (Foster City, CA, USA). All other reagents were purchased from VWR International (Bridgeport, NJ, USA) and used as received.

### 2.2. Participant Recruitment

Women (Test 1: *n* = 114 and Test 2: *n* = 120) were recruited as described elsewhere [17,29] to evaluate prototypes *ex vivo* (in their hands) at the Sensory Evaluation Center at Penn State. Inclusion criteria included: (a) female; (b) between 18 and 55 years of age; (c) reported having had vaginal sex with a man in the last 12 months; (d) were willing to manipulate prototypes with their hands and evaluate them using a computer-guided assessment in an isolated test booth. All procedures were approved by the Pennsylvania State University Institutional Review Board (protocol #36943, approved 25 April 2013). Participants provided informed consent, and were reimbursed for their time.

### 2.3. Sample Design and Preparation

**Figure 1:**
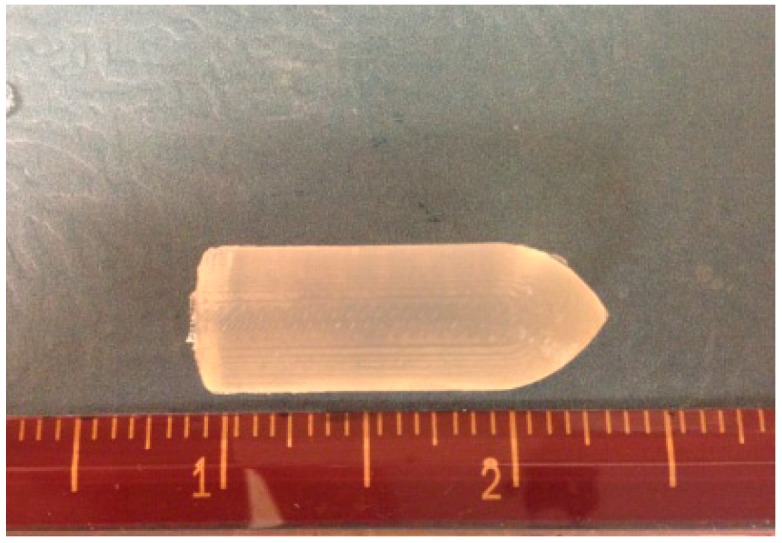
Bullet-shaped suppositories of size 3 mL.

Based on previous acceptability studies, 4 firmness levels ranging in G’ from 250 to 25,000 Pa (25 °C) were selected [15]. To prepare gels of constant firmness with different elongation properties, the types and amounts of ι- and κ-carrageenan were varied, as was the amount of potassium chloride (Table 1). Gels were prepared by mixing the dry ingredients (carrageenan and KCl) in deionized water and holding the mixture at 85 °C for at least 1 h. The gel was stirred intermittently till it formed a homogenous dispersion. Prior to preparing the suppositories, the gel was characterized in a rheometer to confirm the storage modulus closely (within 10%) matched the desired value (Table 1) at 25 °C. Small deformation rheological measurements were performed at a frequency of 1 Hz and strain of 1% on a strain-controlled oscillatory rheometer (ARES, TA Instrument, New Castle, DE, USA) using a cone and plate geometry (probe diameter = 25 mm, cone angle = 5.73°). The gel was loaded between the plates when hot (80 °C) and the edges sealed with a light coating of mineral oil to prevent moisture evaporation. Data were recorded first on cooling to 15 °C, followed by heating to 60 °C at a rate of 5 °C per minute. Bullet shaped suppositories (Figure 1) were prepared by filling plastic syringes with the hot gel and injecting it into acrylonitrile butadiene styrene molds, followed by cooling in a refrigerator (4 °C) for 15 min and holding at room temperature for at least 2 h in sealed glass vessels to allow the gels to set [17,29]. Samples were presented in 0.75 oz Solo transparent plastic cups (Solo Cup Company, Urbana, IL, USA), kept at 25 °C with the lids sealed tightly until evaluated.

**Table 1:** Composition of brittle and elastic gels used to prepare suppositories.

Nominal Storage Modulus (G’) (Pa)	Brittle Formula			Elastic Formula		
	κ (% *w*/*v*)	ι (% *w*/*v*)	KCl (M)	κ (% *w*/*v*)	ι (% *w*/*v*)	KCl (M)
250	0.5	0	0.025	0	2	0.05
2,500	1.25	0	0.04	0.5	3	0.05
12,500	2	0	0.05	1.25	3	0.05
12,500 with TFV	2.5	0	0.05	2	4	0.05
25,000	2.5	0	0.05	2	3	0.05

Antiretroviral drug tenofovir (TFV).

κ-Carrageenan gels are relatively brittle *vis-à-vis* those containing ι-carrageenan. For convenience, we refer to the samples as either “brittle” or “elastic” in the remainder of the manuscript, but these names were never used with participants, who only referred to products using random 3-digit blinding codes.

### 2.4. Sample Evaluation

Participants were asked to watch a 90 s video in which a medical professional demonstrates how a participant should evaluate a prototype in her hand [17,29]. Participants were instructed to: (1) Take the sample and put it into her non-dominant hand; (2) Gently stroke the sample with the index finger of her dominant hand; (3) Put the sample between her fingers and pinch gently (hand not specified verbally; shown as dominant hand in video); (4) Finally hold the sample between her fingers and imagine she was trying to insert the sample into her vagina (hand not specified). After watching the video, participants were provided with a consent form to read. Women who wished to participate were assigned individual ID codes and sent to the testing area.

#### 2.4.1. Sample Evaluation for Test 1

Participants were presented with three pairs of samples: Each pair consisting of the brittle and elastic suppositories of the same storage modulus (G’ = 250, 2500 or 25,000 Pa). For each pair of brittle and elastic suppositories, participants were asked to select their preferred sample in a forced-choice task; they were also provided with an open-ended comment box to provide reasons for their selection if they choose to.

#### 2.4.2. Sample Evaluation for Test 2

Based on the results of Test 1 (see below), the preferred samples were selected and compared to an additional intermediate firmness level with storage modulus G’ = 12,500 Pa, so a total of four suppositories were evaluated. Participants rated their imagined *ease-of-insertion* and *willingness-to-try* on separate 100-point visual analog scales for each of the suppositories. After the individual ratings were obtained, women were asked to rank the four suppositories in order from most to least preferred.

In both tests, participants provided self-report of their age, race, ethnicity, education, marital status, number of vaginal deliveries and vaginal product usage after product evaluation was complete. The majority were married, college-educated white women. Data on prior experience with other vaginal products, collected using a check all that apply question, is summarized in Table 2.

**Table 2:** Prior vaginal product usage from a check that all apply question (column totals may exceed the number of participants).

Vaginal Products	Test 1 (*n* = 114)	Test 2 (*n* = 120)
Vaginal contraceptive products such as Nuvaring	5	4
Spermicidal gels and films	3	5
Yeast infection medicines such as Vagisil and Monistat	26	27
Douche	5	7
Menstruation products such as tampons	71	85
Lubrication products such as KY gels, liquibeads and Vitamin-E suppositories	39	40
Decline to answer	13	13

### 2.5. Mechanical Characterization of Elongation Properties of the Gels

To characterize the large-deformation rheological properties of brittle and elastic gels, the gels were molded into oval rings (outer length of oval = 5.90 cm, center-to-center length = 2.48 cm, radius of outer arc = 1.71 cm, width of gel leg = 1.03 cm, depth of gel = 1.0 cm) and stretched in tension at 5 mm/s on two 1.4 cm aluminum dowel pins (Figure S1A) attached to a TAXT2i Texture Analyzer (Stable Micro Systems, Halsemere, Surrey, UK) [30]. Eight to ten measurements of tensile force and deformation at fracture were made for each gel type and firmness. Tensile force and deformation was also compared with gels prepared with and without TFV to study the effect of TFV addition on the elongation properties. Gels with TFV were prepared with additional amounts of carrageenan as compared to gels without TFV to obtain the same G’ value (12,500 Pa) (Table 1). Dowel pins and the contacting surfaces of the ring were lightly coated with mineral oil to reduce friction. The force and deformation at fracture were measured to calculate the stress at fracture (σ) and strain at fracture (ε) using the equations:

(1) $$\text{σ}=\frac{F}{2A_{0}}\left(1+\frac{2\Delta L}{C_{a}}\right)$$

and

(2) $$\in =\ln\left(1+\frac{2\Delta L}{C_{a}}\right)$$

where: σ is the true stress at fracture (g/m^2^); *F* is the force at fracture (g); *A*_0_ the initial cross sectional area (m^2^); Δ*L* the change in leg length (m); *C*_a_ the average circumference (m); and ϵ the Hencky or true strain at fracture.

As fracture behavior might be altered by exposure to vaginal fluids, we quantified the compression force required to fracture spherical suppositories (size 3 mL) that had been soaked in 5 mL vaginal simulant fluid (VSF) at 37 °C with constant shaking. Compression force was measured at 0, 2, 6 and 24 h using parallel plates and the TAXT2i Texture Analyzer (Figure S1B). Compression force was also compared between gels prepared with and without TFV to study the fracture behavior of the suppositories in the presence of TFV. The vaginal simulant fluid was prepared as described by Owen and Katz [31].

### 2.6. Characterization of Drug Release

The rate of release of the antiretroviral drug tenofovir (TFV) was determined for spherical suppositories made using the brittle and elastic gel formulations. Gels with G’ = 12,500 Pa were selected for the drug release studies, as this firmness was the most favored in Test 2 (results below). Based on the dose of TFV employed in clinical trials with 4 mL of 1% TFV gel [1], each suppository was prepared as previously described [17], with the addition of 40 mg of TFV. The acidic pH of TFV in water (pH = 3–4) disrupted carrageenan gel formation, as the pK_a_ of carrageenan is 4.9. Therefore, the required concentration of TFV was dissolved in deionized water and the pH of the TFV solution was adjusted to 7.2–7.4 by addition of sodium hydroxide. Gels were then prepared as described above and characterized in a rheometer (see above) to confirm the storage modulus closely (within 10%) matched the desired value of G’ = 12,500 at 25 °C. Spherical suppositories were prepared by filling plastic syringes with the hot gel and injecting into molds, followed by cooling and storing as described above. Dissolution studies were performed (a) using USP apparatus 2 (basket apparatus) with 100 mL glass vessels or (b) in a shaking incubator with 50 mL screw-cap test tubes. Studies were run at 150 rpm, 37 °C with 80 mL VSF for the basket apparatus and 5 mL VSF for the shaking incubator. Dissolution studies with 5 mL VSF were designed to better mimic the vaginal environment, as the average volume of vaginal fluid obtained from healthy donors has been shown to vary in the range of 0.5–8 mL/day [32,33,34].

### 2.7. Statistical Analyses

Data were analyzed using JMP v9.0.2 (Cary, NC, USA). For Test 1, preference for brittle and elastic suppository was compared using a binomial test with a *p* value less than 0.05 considered significant. The effect of gel type and storage modulus on the imagined *ease-of-insertion* and *willingness-to-try* was tested via ANOVA, with participant as a random effect and suppository type as a fixed effect. Tukey’s Honest Significant Difference (HSD) was used for *post hoc* comparisons with *p* < 0.05 considered significantly different. Similarly, the effect of suppository type and storage modulus on gel compression and elongation was tested via ANOVA. The least square means (L.S.M.) were then compared across different suppository types or across different G’ values separately using Tukey’s HSD test with *p* < 0.05 considered significantly different. For the TFV release data in water VSF mean and standard error were calculated (*n* = 7) for each time point. For comparing the initial rates of diffusion (0–2 h) across different VSF volumes and suppository types, the slopes for individual samples were computed from the release data. The least square means (L.S.M.) of slopes were calculated and compared using Tukey’s HSD test with *p* < 0.05 considered significantly different.

## 3. Results

### 3.1. Women’s Preferences in Test 1 (Brittle Versus Elastic at Constant Firmness)

**Figure 2:**
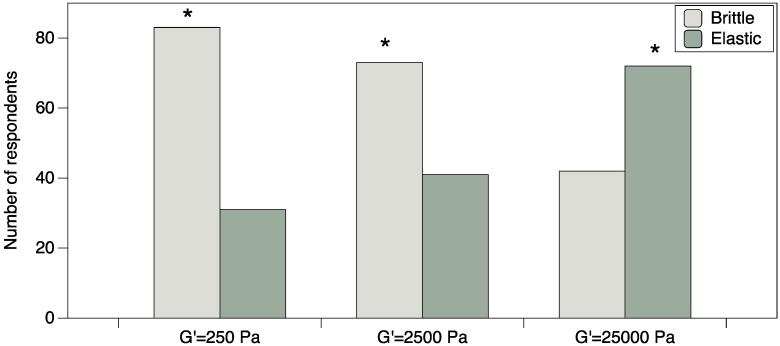
Elasticity and Firmness interact to influence women’s preference. At the lower firmness levels, brittle samples are preferred, whereas at higher firmness the elastic sample is preferred. Asterisks (*) indicate a significant difference for the paired comparison using a binomial test (*p* < 0.05).

To determine the preference for brittle *versus* elastic suppositories at each firmness level, 114 women were asked to select their preferred sample from three pairs of suppositories at G’ of 250, 2500 and 25,000 Pa (see Figure 2). For the 250 Pa G’ prototypes, women preferred brittle over elastic suppositories (*p* < 0.0001) in a forced-choice task. However, in the open-ended comments, many women commented they did not like either suppository in the pair, as they were both too soft. For the 2500 Pa G’ prototypes, women preferred brittle over elastic suppositories (*p* = 0.0035) in a forced-choice task. In the comments, fewer women expressed dislike for either suppository in the pair, suggesting this was a more favorable firmness level. For the 25,000 Pa G’ prototypes, women preferred elastic over brittle suppositories (*p* = 0.0064) in a forced-choice task. In the comments, many women indicated this would be a favorable firmness for insertion. Collectively, women disliked elastic suppositories at G’ 250 or 2500 Pa as they were perceived to be softer than brittle samples at the same G’, flimsy, easily breakable, and thus difficult to insert. However at G’ of 25,000 Pa, the elastic suppositories seemed like a good combination of firmness and softness, which would translate to comfort inside the body, yet be firm enough for easy insertion. Participants made comments such as “soft yet firm, feels as though [it] would be easily placed” and “just right amount of firmness and flexibility”. The brittle suppositories at this high G’ (25,000) seemed too hard, which might cause discomfort upon insertion, as reflected in participant comments like “Too hard—I can’t imagine what that would feel like inside me”.

Based on our earlier qualitative data from in-depth focus groups, prior vaginal product use may influence women’s perception as well as preference for the suppositories [15]. To quantify this using the data from Test 1, preference for brittle *versus* elastic was tested for independence among users *versus* non-users of Yeast infection medication, Menstruation products and Lubrication products using Chi-square tests. No significant associations were observed at any firmness level.

### 3.2. Women’s Preference from Test 2

**Figure 3:**
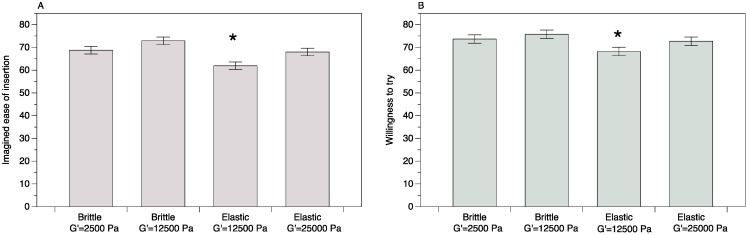
Effect of firmness and texture on (**A**) imagined *ease-of-insertion* and (**B**) *willingness-to-try*, which were measured on a 100 point visual analog scale. Indented semantic anchors at 10 (e.g., “not at all willing”) and 90 (e.g., “extremely willing”) were provided to minimize scale end avoidance bias. Asterisks (*) indicate a significant difference at α = 0.05 (Tukey’s Honest Significant Difference (HSD)).

In our iterative design process, samples at G’ value 2500 and 25,000 Pa (the top 2 firmness levels from Test 1) were compared with elastic and brittle samples at 12,500 Pa. Samples with G’ = 250 Pa were eliminated due to the expressed dislike for both the elastic and brittle samples in the open ended comments from Test 1. All samples except for elastic with G’ = 12,500 Pa scored above 65 (out of 100) for the imagined *ease-of-insertion* (Figure 3A) and above 70 for *willingness-to-try* (Figure 3B) indicating that suppositories formulated in the firmness range of G’ = 2500–25,000 Pa are generally well liked by participants. The Borda counts calculated [35] from the ranking data indicate that brittle suppositories at G’ = 12500 Pa are most preferred by women with brittle samples at G’ = 2500 Pa a close second (Table 3).

**Table 3:** Rank order of samples in order of preference from most preferred (1) to least preferred (4) along with calculated Borda Counts from Test 2.

Rank Order	1	2	3	4	Borda Counts
Brittle (G’ = 12,500 Pa)	40	34	30	16	218
Brittle (G’ = 2,500 Pa)	40	34	24	22	212
Elastic (G’ = 25,000 Pa)	22	31	43	24	171
Elastic (G’ = 12,500 Pa)	18	21	23	58	119

### 3.3. Characterization of Elongation Properties of Brittle and Elastic Gels

Rings prepared from the elastic and brittle gels were stretched and the tensile force and deformation required to fracture the rings was used to calculate the stress (σ) and strain (ε) at fracture (Table 4). The elastic gels withstood greater strain at fracture as compared to brittle samples for all firmness levels tested. The stress at fracture increased linearly with firmness in the range tested for brittle gels, whereas it plateaued between G’ = 12,500 and 25,000 Pa for elastic gels. For both the brittle and elastic gels the strain at fracture is significantly different for G’ = 2500 as compared to G’ = 12,500 or 25,000 Pa. However in the case of brittle gels the strain increased with increasing firmness whereas for elastic gels it progressively decreased with increasing firmness.

**Table 4:** Stress and strain at fracture for brittle and elastic gels at increasing G’ values calculated using force and deformation required to fracture gel rings. Upper case letters denote statistically significant differences (*p* < 0.05) in the stress and strain between the two gel types. Lower case letters denote statistically significant differences (*p* < 0.05) in the stress and strain across the different G’ values for the same gel type.

Storage Modulus	Brittle		Elastic	
G’ (Pa)	Stress (σ) (g/m^2^)	Strain (ε)	Stress (σ) (g/m^2^)	Strain (ε)
2500	42.72 ^A,a^	0.088 ^P,f^	65.76 ^B,d^	0.313 ^Q,j^
12,500	155.96 ^C,b^	0.139 ^R,g^	158.23 ^C,e^	0.233 ^S,k^
25,000	360.14 ^E,c^	0.16 ^T,g^	157.59 ^F,e^	0.21 ^U,k^
**Storage Modulus**	**Brittle without TFV**		**Brittle with TFV**	
**G’ (Pa)**	**Stress (σ) (g/m^2^)**	**Strain (ε)**	**Stress (σ) (g/m^2^)**	**Strain (ε)**
12,500	155.96 ^G^	0.139 ^K^	192.93 ^H^	0.152 ^L^

**Figure 4:**
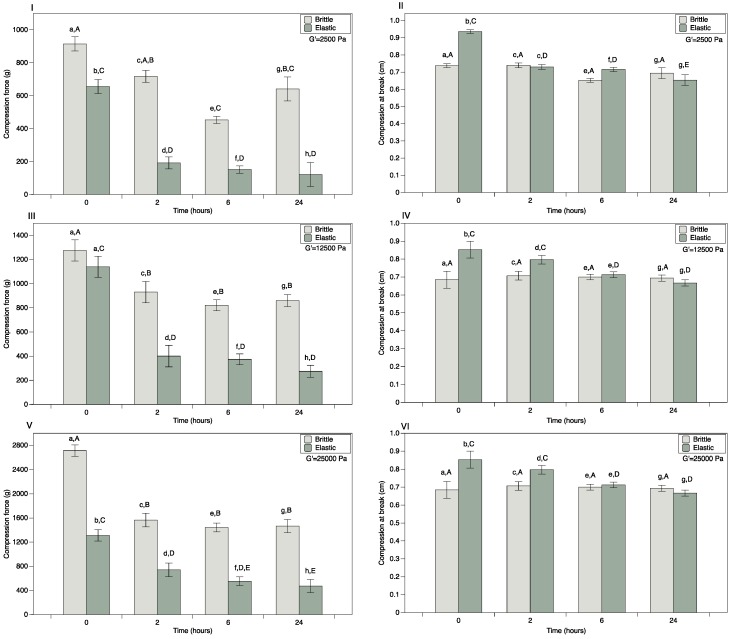
Compression force required to break spherical suppositories and compression at break after suppositories have been in contact with vaginal simulant fluid (VSF) from 0–24 h. Compression force required to break the ovule and the deformation at fracture are plotted for G’ = 2500 Pa (**I**,**II**), G’ = 12,500 Pa (**III**,**IV**) and G’ = 25,000 Pa (**V**,**VI**). Lower case letters denote statistically significant differences between brittle and elastic suppositories at the specific time point. Upper case letters denote statistically significant differences (*p* < 0.05) between time points for the same suppository type (brittle and elastic).

Ideally, the suppositories will break down within the body releasing the medication. Since the gel is designed to mimic the vaginal mucous secretions once it erodes, it can be covertly eliminated from the body. Measuring the compression force required to fracture the ovules enables quantification of ovule softening over time. Consistent with the elongation properties, the force required to fracture the ovules in compression was significantly higher for brittle as compared to elastic ovules (Figure 4 I,III,V). The force required to fracture both brittle and elastic ovules in compression generally decreased on contact with VSF for the first 2 h with much slower decrease over the next 24 h (Figure 4 I,III,V). The deformation at fracture did not vary over time for brittle suppositories; however for the elastic suppositories the deformation at fracture decreased over time (Figure 4 II,IV,VI). Brittle suppositories prepared at G’ = 12,500 Pa with TFV fractured with significantly lower force and distance in compression as compared to suppositories without TFV (data not shown).

### 3.4. Characterization of Drug Release

**Figure 5:**
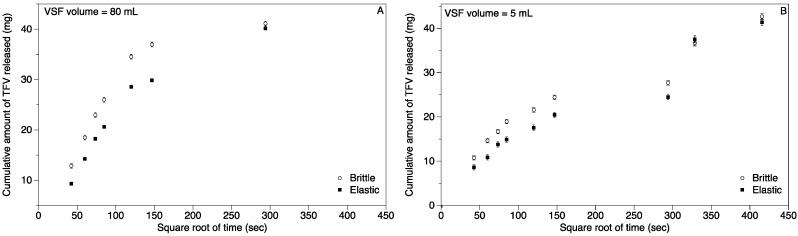
Effect of gel type on release of antiretroviral drug tenofovir (TFV) from spherical carrageenan suppositories in (**A**) 80 mL VSF; (**B**) 5 mL VSF. Release of TFV by diffusion from suppositories of size 3 mL loaded with 40 mg TFV each was studied for 24–48 h. Plotted are mean and standard error (*n* = 7) of the cumulative amount of TFV released.

The release of tenofovir (TFV) loaded into spherical suppositories prepared from brittle and elastic gels at G’ = 12,500 Pa was characterized over 24–48 h (depending on VSF volume). For diffusion into 80 mL VSF, 65% of the encapsulated TFV was released within the first 2 h, and over 90% was released within 6 h (Figure 5A) from the brittle suppositories. In contrast, for the elastic suppositories in the same VSF volume, 50% of the encapsulated TFV was released within the first 2 h, and 75% was released within 6 h (Figure 5A). All of the TFV within the brittle and elastic suppositories was released within 24 h (Figure 5A). For diffusion into 5 mL VSF from brittle suppositories 45% of the encapsulated TFV was released within the first 2 h, 60% within 6 h and 70% in 24 h (Figure 5B). For elastic suppositories releasing in 5 mL VSF medium, 35% of the encapsulated TFV was released within the first 2 h, 50% within 6 h and 60% in 24 h (Figure 5B). Previous studies with 5 mL dissolution medium have indicated that the release plateaus at 24 h [36]. Recognizing the limitation of insufficient sink conditions, for the 5 mL dissolution studies the dissolution medium was replenished with fresh VSF at 24 h to stimulate additional release. All of the TFV within both the brittle and elastic suppositories was released in the next 24 h (Figure 5B).

The initial TFV release profile in VSF (0–2 h) showed a linear relationship between the cumulative release of TFV and the square root of time. The slope of the linear portion of the line (0–2 h) was used to calculate the rate of TFV diffusion (Table 5). There was a significant difference in the rate of diffusion between the brittle and elastic suppository types for both dissolution volumes (80 and 5 mL) with a higher diffusion rate for brittle suppositories. Also for both suppository types, the diffusion rate in 80 mL VSF was higher than in 5 ml VSF (Table 5). 

**Table 5:** Suppository type influences rate of TFV diffusion from semisoft carrageenan suppositories (G’ = 12,500 Pa). Calculated initial (0–2 h) *in vitro* release rates for TFV into vaginal simulant fluid (VSF) for elastic and brittle suppositories. Upper case letters denote statistically significant differences (*p* < 0.05) in the diffusion rates between the two volumes of dissolution medium for the same type of suppository. Lower case letters denote statistically significant differences in the diffusion rates across the two suppository types for the same VSF volume.

Suppository Type	L.S.M. Release Rate in mg/(s½)	
	80 mL VSF	5 mL VSF
Brittle	0.309 ^A,a^	0.220 ^B,c^
Elastic	0.246 ^C,b^	0.178 ^D,d^

L.S.M., least square means.

## 4. Discussion

Microbicides are an active area of research in the field of HIV prevention; they have the particular advantage of being a woman-controlled method of prevention [2]. While shown to be biologically efficacious, a number of the microbicides have failed to demonstrate efficacy in clinical trials, largely due to poor compliance by study participants [4,6]. Compliance may depend on women’s perception of efficacy as well as product acceptability hence, research efforts are being directed to determine product features affecting women’s perception and liking of the microbicide candidates [12,13,15,17,29]. We have developed carrageenan-based soft-gel suppositories with varying physical characteristics to determine favorable product characteristics that drive acceptability and willingness to try. In our previous development efforts, the different design parameters that we varied in our prototypes are firmness (5 levels), size (3 levels) and shape (6 distinct shapes) [15]. In this iterative design process, we developed two types of carrageenan gels with the same storage modulus but different elongation properties to determine if the extensibility plays a role in women’s preference and willingness to try.

Previous optimization efforts indicated that ease of insertion is a critical factor governing women’s willingness to try this product [15,17], and that the rheological properties, both small and large deformation, may play a role in the imagined ease of handling and insertion.

The current study investigated the role of large deformation properties on preference, willingness to try, and imagined ease of insertion. Our initial hypothesis was that women would prefer the elastic samples to brittle samples in the lower firmness range, as they would not break as easily during handling and insertion as the brittle ones. However, women preferred the brittle samples at G’ = 250 and 2500 Pa and preferred the elastic samples at G’ = 25,000 Pa. Contrary to our initial hypothesis, the elastic samples were perceived to be softer, flimsy, easily breakable and difficult to insert at lower firmness. Based on the results of the physical compression test, the elastic suppositories with G’ = 2500 Pa could withstand greater deformation prior to fracturing however the force required to fracture them prior to VSF contact was significantly lower than brittle samples. Thus the brittle samples were perceived to be sturdier, which may explain why women preferred brittle samples to elastic ones at the lower firmness. Focus group discussions conducted previously established that imagined comfort upon insertion plays a role in women’s preferences, along with imagined ease of insertion [15]. Brittle samples with G’ = 25,000 Pa were preferred in previous work. However when offered the choice here, women preferred the elastic sample at G’ = 25,000 Pa as it retained the desirable quality of being easy to insert and was also perceived soft enough to be comfortable once inserted. Additionally data from focus groups also indicated that color plays a role in women’s preferences, especially since the color of the suppository corresponds to the appearance of the vaginal discharge [15]. The brittle suppositories were slightly clearer in appearance than the elastic ones and this may have influenced women’s preference for brittle suppositories. However with ι-carrageenan (required for elastic gels), higher firmness requires higher concentration and the color/opacity differences although small may be unavoidable. The storage modulus value (G’) has been used for quantifying firmness of the prototypes developed in the laboratory and previous development efforts did not include elongation properties as a design parameter. Present results provide support for combining the storage modulus with large deformation testing to fully characterize the gels during preclinical development.

In our second test, when an intermediate firmness level was evaluated alongside the preferred samples from Test 1, the most preferred firmness shifted from G’ = 25,000 Pa [17,29] to a brittle suppository with G’ = 12,500 Pa. Notably, preferences may be influenced by the other options presented within the choice set [37]. The gels of varying firmness prepared previously [17,29] had varied ratios of κ- and ι-carrageenan; thus, firmness was partially confounded with elongation properties. Based on comments from focus group participants [15], here we prepared gels with the same storage modulus yet different elongation properties to assess their effect on preference to refine our understanding of women’s preferences. The ranking data indicated that brittle suppositories with G’ = 12,500 Pa were most preferred; still all the samples except elastic at G’ = 12,500 Pa were highly rated for *ease-of-insertion* and *willingness-to-try*, suggesting brittle samples in the range G’ = 2500–12,500 Pa and the elastic samples with G’ = 25,000 Pa were well accepted.

Our design process simultaneously optimizes different physical characteristics believed to drive acceptability, and performance parameters such as drug release and residence time in the vagina. Critically, producing microbicides in a wide range of firmness that are all highly acceptable may allow for better optimization of the drug release profile. The release of TFV from the carrageenan matrix depends on various factors such as drug loading, density of the carrageenan gel, partition coefficient of the drug between water and carrageenan, and molecular size of the drug. Among these factors, the density of the gel depends on the total carrageenan concentration [38], which greatly varies between brittle and elastic gels at the same firmness level. The elastic gels at G’ = 12,500 Pa have twice the total carrageenan concentration as compared to the brittle gels, thus slowing down the diffusion from the elastic gel suppositories. The initial rate of diffusion is higher in 80 mL VSF as compared to 5 mL VSF for both the brittle and elastic suppositories. The mass transport of TFV from the carrageenan matrix depends on the concentration of TFV in the saturated zone surrounding the suppository. The lower volume of VSF (5 mL) results in faster saturation in the surrounding medium as compared to 80 mL, thus slowing down TFV release. Due to insufficient sink condition with 5 mL VSF, TFV release plateaus at 24 h, and only replenishing with fresh VSF stimulates additional release. Since TFV prophylaxis works by diffusing into the vaginal walls, this will help maintain a concentration gradient across the suppository, vaginal lumen and vaginal tissue [3]. The vaginal discharge in women is composed of several components such as vulvar secretions, cervical mucus, and endometrial fluid, and its amount varies based on several factors such as menstrual cycle, hormone levels, and sexual arousal [39]. Due to physiologic processes, there is regular flushing of the fluids in the vagina, which may help maintain a concentration gradient that allows TFV diffusion into the vaginal lumen.

While characterizing the elastic properties of the gel, the strain required to fracture rings was lower for brittle gels as compared to elastic gels. However, in the case of brittle gels, the strain increased with increasing firmness whereas for elastic gels it progressively decreased with increasing firmness. This can be explained in part by the varying ratio of ι- to κ-carrageenan used to prepare elastic gels of increasing firmness. Elastic gels of G’ = 2500 Pa were prepared at an ι:κ ratio of 6:1 and ι is reported to form elastic gels [40], hence it can withstand the highest fracture strain. Addition of increasing amounts of κ-carrageenan to prepare elastic gels with increasing firmness led to more brittle gels (though not as brittle as κ-carrageenan alone).

Upon contact with vaginal fluid, our suppositories are intended to break down and be eliminated with vaginal mucous secretions. To test the disintegration or matrix softening of carrageenan in the vaginal environment, suppositories were soaked in VSF for 24 h at body temperature with continuous stirring. The current prototypes do not completely disintegrate in contact with VSF. However, there is a significant decrease in the amount of force required to fracture ovules upon soaking in VSF for 2 h. Since this force does not continually decrease with time, efforts are on-going to reformulate gels to aid disintegration in the vagina.

## 5. Conclusion

In the current paper we show that different physical properties of the gel including the small and large scale deformation properties influence women’s preferences as well as willingness to try microbicide prototypes. Both storage modulus and elongation properties affect women’s perception of the firmness, which in turn influences their imagined ease-of-insertion. Ease of insertion greatly influences their willingness to try the product, as the product is only good if inserted consistently, correctly and without hassle. Understanding the factors that affect women’s willingness to use a specific drug delivery product is a critical step in the design of an effective microbicide.

## Supplementary Files

Supplementary File 1
