# Supplementary material for: Firmness Perception Influences Women’s Preferences for Vaginal Suppositories
Source: Pharmaceutics. 2014 Sep 10;6(3):512–29. doi: 10.3390/pharmaceutics6030512 (PMC4190533; doi:10.3390/pharmaceutics6030512)
Supplement: Supplementary File 1 [file pharmaceutics-06-00512-s001.docx]

**Supplementary Information**

**Figure S1.** (**A**) Setup to test the force and deformation required to fracture gel rings, using TAXT 2 Texture analyzer; (**B**) Setup to test the force in compression required to fracture spherical suppositories using TAXT 2 Texture analyzer.

|  |  |
| --- | --- |

© 2014 by the authors; licensee MDPI, Basel, Switzerland. This article is an open access article distributed under the terms and conditions of the Creative Commons Attribution license (http://creativecommons.org/licenses/by/3.0/).
